# Oxidative Stress Biomarkers in Cystic Fibrosis and Cystic Fibrosis-Related Diabetes in Children: A Literature Review

**DOI:** 10.3390/biomedicines11102671

**Published:** 2023-09-29

**Authors:** Anca Daniela Pinzaru, Cristina Maria Mihai, Tatiana Chisnoiu, Alexandru Cosmin Pantazi, Vasile Valeriu Lupu, Mustafa Ali Kassim Kassim, Ancuta Lupu, Elena Grosan, Ahmed Zaki Naji Al Jumaili, Irina Ion, Gabriela Stoleriu, Ileana Ion

**Affiliations:** 1 Department of Pediatrics, Faculty of Medicine, “Ovidius” University, 900470 Constanta, Romania; 2 Department of Pediatrics, County Clinical Emergency Hospital of Constanta, 900591 Constanta, Romania; 3 Department of Pediatrics, “Grigore T. Popa” University of Medicine and Pharmacy, 700115 Iasi, Romania; 4 Faculty of Medicine, “Ovidius” University, 900470 Constanta, Romania; 5 National Institute of Diabetes, Nutrition and Metabolic Diseases “N.C. Paulescu”, 020475 Bucharest, Romania; 6 Faculty of Medicine and Pharmacy, “Dunarea de Jos” University of Galati, 800008 Galati, Romania

**Keywords:** cystic fibrosis, diabetes mellitus, oxidative stress, children

## Abstract

The most common inherited condition that results in death, particularly in those of Caucasian heritage, is cystic fibrosis (CF). Of all the young adults diagnosed with cystic fibrosis, 20% will develop hyperglycemia as a complication, later classified as a disease associated with cystic fibrosis. Impaired insulin secretion and glucose intolerance represent the primary mechanisms associated with diabetes (type 1 or type 2) and cystic fibrosis. Oxidative stress represents the imbalance between oxygen-reactive species and antioxidant defense mechanisms. This pathogenic mechanism is vital in triggering other chronic diseases, including cystic fibrosis-related diabetes. It is essential to understand oxidative stress and the significant impact it has on CFRD. This way, therapies can be individually adjusted and tailored to each patient’s needs. This review aims to understand the connection between CFRD and oxidative stress. As a subsidiary element, we analyzed the effects of glycemic balance on complications and their evolution over time, providing insights into their potential benefits in mitigating oxidative stress-associated complications.

## 1. Introduction

Cystic fibrosis (CF) is the predominant hereditary disease-causing fatality, especially among individuals of Caucasian descent. It is an autosomal recessive disease that affects approximately one in every 3000 live births [1]. The gene responsible for cystic fibrosis is a regulator called the cystic fibrosis transmembrane conductance regulator (CFTR) gene. As a result, this dysfunctional protein interferes with the regular transportation of chloride ions, disturbing the movement of epithelial lining fluid (mucus) in various organs [2].

Cystic fibrosis-related diabetes (CFRD) represents a frequently encountered complication in individuals with CF, affecting approximately 20% of adult CF patients [3]. The easiest way to define cystic fibrosis-related diabetes is by hyperglycemia and its consequences [4]. 

A loss of equilibrium between oxygen-reactive species and antioxidant defense systems characterizes oxidative stress. This pathogenic mechanism is vital in triggering other chronic diseases, including cystic fibrosis-related diabetes [5]. ROS comprises superoxide anion or hydrogen peroxide, which are defined as byproducts of normal cellular metabolism involved in various physiological processes [6]. Lipids, proteins, and even the DNA structure can be affected by the excess production of ROS and a reduced antioxidant capacity. All of this leads to tissue damage and much faster disease progression [7]. The underlying mechanisms in CF that result in oxidative stress are multifactorial. The defective CFTR protein disrupts ion transport, leading to altered intracellular ion concentrations, impaired pH regulation, and increased activation of important cells, represented especially by neutrophils or macrophages, which produce ROS in the inflammatory response [8,9]. CFTR dysfunction impairs the transport of glutathione, a key antioxidant molecule, further compromising the antioxidant defenses in CF cells [10]. Different molecular mechanisms can define the dysfunction of the CFTR [10]. Due to increased oxidative stress, these cumulative effects affect various CF-associated organs. The onset and advancement of CFRD are connected to oxidative stress implications. The first notice modification is represented by the altered pancreatic beta-cell, followed by apoptosis. Impaired insulin signaling pathways and insulin resistance are the second main adjustments [11,12]. Furthermore, chronic inflammation, commonly observed in both CF and diabetes, contributes to increased ROS production and exacerbates oxidative stress [13]. The relationship linking oxidative stress and impaired glucose metabolism in CFRD underscores the importance of studying oxidative stress biomarkers as potential diagnostic and prognostic tools. Understanding the impact of oxidative stress in CF and CFRD is crucial for developing targeted therapies and improving patient outcomes.

Moreover, investigating the effects of diabetes therapies on oxidative stress can provide valuable insights into the mechanisms underlying their efficacy and potential for reducing disease burden. This review aims to understand the connection between CFRD and oxidative stress. As a subsidiary element, we analyze the effects of glycemic balance on complications and their evolution over time, providing insights into their potential benefits in mitigating oxidative stress-associated complications. The main connection analyzed is between diabetes and imbalanced oxidative stress, shedding light on the intricate molecular pathways connecting oxidative stress with the development of diabetes. Additionally, we examine the benefits of diabetes therapies on oxidative stress biomarkers, providing insights into their potential benefits in mitigating oxidative stress-associated complications.

## 2. Biomarkers of Oxidative Stress

**Figure 1:**
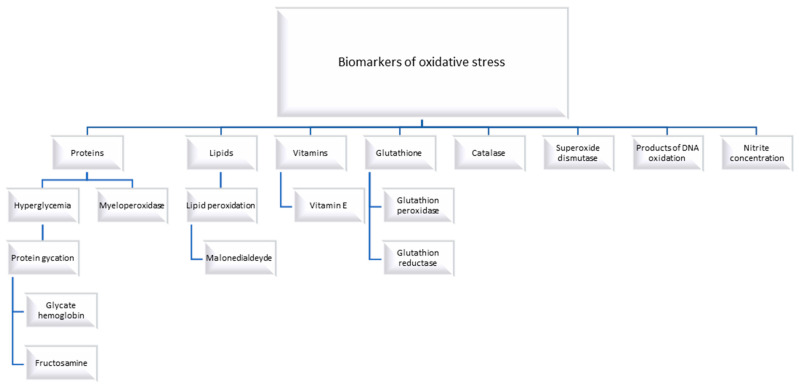
Biomarkers of oxidative stress in diabetes [15,16,17].

Oxidative stress assessment can be made by testing endothelial function and measuring reactive oxygen and nitrogen species, oxidative damage makers/expression levels of reactive oxygen species-producing enzymes, and the level of antioxidants [14]. Biomarkers of oxidative stress include proteins, lipids, vitamins, glutathione, catalase, products of DNA oxidation, nitrite concentration, and superoxide dismutase can be measured in diabetic individuals [15,16,17] (Figure 1).

In type 1 and type 2 diabetes, protein oxidation and lipid peroxidation indicators are markers for oxidative stress [18]. Furthermore, these patients have elevated levels of 8-iso-prostaglandin F2α, which results from the non-enzymatic peroxidation of arachidonic acid in the membrane phospholipids [14,15]. Increased values of 8-iso-PGF2α are alarming signs of atherosclerosis [19,20,21,22]. N. Colomo et al. conducted a study involving diabetic children and adolescents. It established there are high urinary levels of 8-iso-PGF2α in type 1 diabetes individuals [21,22,23]. Anita Morandi et al. concluded that type 1 pediatric diabetes patients have higher concentrations of derivatives-reactive oxygen metabolites (D-ROMS) than healthy individuals [24]. 

However, not only hyperglycemia plays a role in triggering oxidative stress but also glucose variability. Independent of glycemic control, glycemic variations are considered a defining element in accelerating oxidative stress and accentuating its teratogenic effect [24,25].

Type 2 diabetes mellitus (T2DM) is considered a defining pathogenic element in accentuating oxidative stress, Ref. [21] leading to inflammation, an increase in reactive oxygen species, and a decrease in antioxidant levels, ultimately accentuating the oxidative stress. These harmful factors contribute to endothelial and vascular injuries [26]. Tauman et al. evaluated the contribution of obstructive sleep apnea to oxidative stress in obese children with and without diabetes. Sleep apnea was associated with higher lipid peroxidation levels, insulin resistance, and hypertension and, therefore, with an increased oxidative stress level [25]. Table 1 summarizes the studies on oxidative stress biomarkers.

## 3. Oxidative Stress in Cystic Fibrosis

Cystic fibrosis, a hereditary condition inherited in an autosomal recessive manner, originates from genetic mutations within the CFTR gene. These mutations lead to compromised function of the CFTR protein [28,29]. Altered ion transport encountered after an impaired CFTR protein results in thickened mucus, impaired mucociliary clearance, and chronic bacterial infections [2]. A significant pathway to morbidity or, even worse, mortality is the progressive decline in lung function [30].

The pathogenesis of CF involves multiple mechanisms that contribute to oxidative stress. An important factor is the dysregulated inflammation seen in CF lungs [31]. The chronic airway inflammation noticed in patients with cystic fibrosis induced by the infiltration of neutrophils, macrophages, and other immune cells, leads to the release of excessive amounts of reactive oxygen species (ROS) as part of the inflammatory response [31,32,33,34]. Vital tissue damage can be induced by ROS-increased production secondary to neutrophils implications [35]. 

Furthermore, the defective CFTR protein disrupts the balance of intracellular ions, resulting in higher levels of intracellular calcium and lower levels of bicarbonate and glutathione [36,37]. Glutathione, a potent antioxidant, protects cells from oxidative stress [38]. CFTR dysfunction leads to impaired glutathione transport, reducing its availability and compromising the antioxidant defenses of CF cells [10]. Iron accumulation is another mechanism leading to oxidative stress in CF [39,40]. The excess iron can participate in the Fenton reaction, generating highly reactive hydroxyl radicals [41]. Several oxidative stress biomarkers have been investigated in CF patients to evaluate the extent of oxidative stress and its potential impact on disease progression. Malondialdehyde (MDA) and F2-isoprostanes are the best biomarkers resulting from lipid peroxidation. They are used as elements to define lipide oxidative damage [42,43,44,45]. Protein carbonylation, which refers to the oxidative modification of proteins, can be measured as an indicator of protein oxidation [46]. As mentioned earlier, cellular antioxidant defense is enforced by glutathione implication [47]. Reduced levels of glutathione or alterations in its redox status can indicate oxidative stress in CF cells [10]. To properly evaluate antioxidant capacity, the most frequent enzymes involved are measured (catalase, superoxide dismutase, and glutathione peroxidase) [46].

Oxidative stress pathogenesis implies several clinical manifestations frequently encountered in CF. Chronic oxidative stress determines the progressive deterioration of lung function, contributing to the development of COPD, asthma, fibrosis, and respiratory failure [48,49]. A recent CF porcine study revealed increased pancreatic oxidative stress markers, including modified proteins and decreased antioxidant enzyme activity [50]. CF islets exhibited elevated oxidative stress, reduced insulin secretion, and impaired secretory function [50]. These conclusions support the idea that oxidative stress could induce secondary insulin deficiency, which was also noticed in patients with cystic fibrosis.

## 4. The Importance of Oxidative Stress in the Pathophysiology of Diabetes

Diabetes, a chronic metabolic disorder, is characterized by persistent hyperglycemia resulting from insufficient insulin production, impaired insulin action, or both [51]. The two major forms of diabetes, T1DM AND T2DM, are differentiated by underlying eti-ological factors. T1DM is characterized by the autoimmune destruction of pancreatic β-cells, resulting in a deficit of insulin. On the other hand, T2DM is linked to insulin resistance and dysfunction of the pancreatic β-cells [51]. The other classes of diabetes include gestational diabetes mellitus (GDM) and diabetes that is induced by or linked to particular circumstances and medical conditions [52]. 

Oxidative stress contributes to the development of diabetes, serving as a pivotal factor in their pathogenesis [53,54]. Studies show that ROS-induced β-cell damage triggers autoimmune responses, culminating in β-cell destruction and insulin deficiency [53,55,56,57]. Chronic overnutrition and physical inactivity accentuate oxidative stress, leading to insulin resistance and impaired secretion [26].

The imbalance between ROS and antioxidant capacity is the defining element in the progression of diabetes [58]. Reactive oxygen species are induced by hyperglycemia, simultaneously reducing the levels of antioxidants [59,60]. Juan Li et al. [61] conducted an analysis focusing on individuals with T2DM diabetes, and the findings revealed a significant correlation between hyperglycemic crises and oxidative stress among diabetic patients. These suggest that in individuals with diabetes, episodes of hyperglycemia are closely associated with heightened oxidative stress. 

Monika Grabia et al. [27], analyzed serum levels of antioxidant enzymes, minerals, and toxic elements to assess their major impact in diabetes progression. The study revealed that individuals with T1DM exhibited a diminished capacity for antioxidant defense within their system, resulting in elevated oxidative stress levels. Morandi et al. [24] conducted a study that found that pediatric patients diagnosed with T1DM exhibited elevated levels of derivatives-reactive oxygen metabolites (D-ROMS) compared to those in good condition. However, it is noteworthy that oxidative stress is not solely triggered by hyperglycemia, as glucose variability also plays a significant role [62]. Consequently, glucose variability might contribute to oxidative stress and its associated atherogenic effects among individuals with T1DM, irrespective of their short-term and long-term average glucose levels [24].

The protein kinase C (PKC) pathway, activated by ROS, contributes to insulin resistance in diabetes [63,64,65]. Similarly, ROS activation of the kappa-light-chain-enhancer of activated B cells (NF-κB) determines the activation of pro-inflammatory cytokine production, exacerbating β-cell damage and insulin resistance [66]. Also, the hexosamine biosynthetic pathway (HBP), influenced by high glucose levels, induces oxidative stress [67]. Oxidative stress, besides being a key factor in diabetes pathogenesis, also contributes significantly to the development of diabetes-related complications. Oxidative stress secondary hyperglycemia determines the usual diabetes complications (retinopathy, nephropathy, neuropathy, and cardiovascular diseases) [68,69,70,71], thus, mitigating oxidative stress might prove beneficial in preventing or slowing the progression of these complications.

Oxidative stress and insulin resistance are correlated through numerous interrelated processes. Firstly, oxidative stress can destroy mitochondria, impairing their function and resulting in dysfunctional β-cells [72]. Furthermore, nuclear transcription factors that are important for the regulation of insulin, such as insulin promoter factor-1, can be inhibited by oxidative stress [73]. Additionally, it may be a factor in the decreased expression of glucose transporter type 4 (GLUT-4), which could compromise the uptake of glucose by the cells [73,74]. The destructive impact on mitochondria by oxidative stress also hampers the availability of energy required for efficient glucose uptake [72,73]. Lastly, the presence of free radicals can impair the insulin signal transduction pathway, further exacerbating insulin resistance [65]. The intricate and multidimensional relationship between oxidative stress and insulin resistance is shown by these interconnected pathways. Oxidative stress and chronic inflammation form the basis of diabetes’ pathophysiology and its complications [18,73,74]. Persistent hyperglycemia releases free radicals in the β-cells, primarily superoxide anions [24,74].

Multiple mechanisms underlie the relationship between oxidative stress and insulin resistance. These include the destruction of mitochondria by oxidative stress, which results in β-cell dysfunction [72], the inhibition of nuclear transcription factors (such as insulin-promoter-factor 1) [74], the decreased expression of the glucose transporter type 4 (GLUT-4) [74,75], the lack of energy needed for glucose uptake due to mitochondrial destruction [72,74], and the disruption of the insulin signal transduction pathway brought on by free radicals [74]. Excessive reactive oxygen species can cause serious diabetes problems that include the kidneys, eyes, and nerves [74,75,76]. Growth factors and pro-inflammatory cytokines are produced in larger quantities when reactive oxygen species are produced excessively. This process and additional metabolic pathways brought on by hyperglycemia control aberrant angiogenesis, tissue ischemia, and vasoconstriction [74]. Hyperglycemia causes the mitochondria to produce reactive oxygen species and reduce antioxidants. After analyzing type 2 diabetic patients, Juan Li found a substantial correlation between hyperglycemic crises and oxidative stress in diabetic patients [61,77,78].

### Diabetes Therapy and Oxidative Stress

The management of CFRD has traditionally been centered on correcting hyperglycemia through administering insulin combined with dietary modifications and physiotherapy [79]. New approaches have been incorporated in recent years, including oral hypoglycemic agents and incretin-based therapies [80,81,82]. However, there are no conclusive data to promote the use of these medications. Some evidence suggests that conventional and novel CFRD therapies can influence oxidative stress biomarkers. For instance, by reducing the generation of ROS, metformin therapy lowers blood glucose levels and oxidative stress [25]. By increasing antioxidant enzymes and lowering inflammatory cytokine levels, incretin-based treatments (e.g., glucagon-like peptide-1 (GLP-1) or dipeptidyl peptidase-4 (DPP-4) inhibitors) have shown antioxidative effects [83]. Exercise has been regularly proven to increase antioxidant capacity, decrease ROS production, and enhance cellular insulin sensitivity [84].

Moreover, physical activity substantially modifies the relationship between glycemic fluctuation at night and oxidative stress during the day [73,85]. Moreover, incorporating a nutritious diet reduces inflammation and oxidative stress and enhances cellular responsiveness to insulin. Therefore, including natural, antioxidant-rich sources is crucial in mitigating oxidative stress [73]. In research of 60 people recently diagnosed with T1DM, Wang et al. analyzed the relationship between the initial insulin dosage and the degree of oxidative stress. Moreover, physical activity substantially modifies the association between glycemic fluctuation at night and oxidative stress during the day [86]. The findings indicated that higher insulin doses initially resulted in a rapid decline in blood glucose levels. However, after 2 and 3 weeks, the disparity in glucose reduction became less discernible as lower, moderate, and high insulin doses exhibited similar effectiveness. Notably, the amount of oxidative stress was constant, independent of the amount of insulin given. Thus, reducing oxidative stress in CFRD management can have significant clinical implications.

## 5. Cystic Fibrosis-Related Diabetes

### 5.1. Definition and the Importance of the Subject

CFRD is an extrapulmonary complication of CF resulting from abnormal glucose metabolism [87]. CFRD shares characteristics with both T1DM and T2DM. However, its distinguishing feature of acute pulmonary complications associated with hyperglycemia and catabolic metabolism, along with a relative insulin deficiency, impacts the application of typical diabetes definitions and therapies. As people with CF live longer lives, it is expected that more than half will acquire CFRD during their lifespan, including up to 20% of adolescents. As the number of persons with CFRD grows, diabetes practitioners will become more aware of the disease [88].

### 5.2. Evolution, Comorbidities and Mutations

Among patients with cystic fibrosis, diabetes associated with the disease is the most prevalent non-pulmonary comorbidity [89]. Individuals suffering from cystic fibrosis-related diabetes are prone to greater mortality caused by nutritional status and lung disease exacerbations (more frequently in CFRD) [90]. CFRD prevalence increases with age as β-cell dysfunction and destruction progress [91]. The primary causing factor of cystic fibrosis-related diabetes is insulin insufficiency [89], similar to type 1 diabetes, due to the destruction and impairment of pancreatic cells [92]. Nonetheless, people with CFRD may also exhibit partial insulin resistance. Insulin resistance is more significant when using a glucocorticoid treatment, during acute pulmonary exacerbations, and as people age [89]. The severity of CFTR mutations is correlated with an increased risk of diabetes [89,90,91,92,93]. Insulin exocytosis and pancreatic development are mediated by β-cells, which express CFTR [92]. Insulin insufficiency and cell dysfunction are also caused by the β-cells’ increased susceptibility to oxidative stress [94,95]. CFRT is also expressed in α-cells [94] and, due to its dysfunction, it determines dysregulated glucagon secretion and contributes to glucose intolerance and CFRD pathophysiology [92].

### 5.3. Associated Genetic Factors

The development of cystic fibrosis-related diabetes is influenced by genetic factors, including the factor 7-like 2 (TCF7L2) gene, which has also been associated with type 2 diabetes. This involvement was confirmed by Blackman et al. in 2013. Also, Blackman et al. identified four new loci that are associated with the disease: cyclin-dependent kinase 5 (CDK5), cyclin-dependent kinase inhibitors 2A and 2B (CDKN2A/B), insulin-like growth factor 2 messenger RNA (IGF2BP2), and solute carrier family 26 members 9 (SLC26A9). Additional genes implicated in the development of cystic fibrosis-related diabetes (CFRD) include PM20d1, which is located near the SLC26A9 gene, and PTMA. This gene modifier was found by Aksit et al. [96]. The extent of cystic fibrosis manifestations is directly correlated with the CFTR genotype. 

Furthermore, the F508del mutation (homozygous and heterozygous states) determined impaired glucose metabolism. Furthermore, homozygous individuals for F508del mutations have diminished insulin sensitivity, reduced insulin levels during oral testing, and decreased first-phase insulin responses during intravenous glucose tolerance tests compared to heterozygotes. In addition, several more genetic modifiers are associated with both CFRD and T2DM besides the genes already mentioned that are believed to affect insulin secretion and β- cell function [92].

### 5.4. Glucose Homeostasis in CFRD, Pathogeny of CFRD

The insulin secretion impairment associated with cystic fibrosis determines the hyperglycemia to install. The physiological process of insulin is to be produced by beta-cells of the pancreas and stimulated by glucose load during meals or stress [97]. It is a peptidic hormone synthesized and stored in the Langerhans cells. After secretion, insulin action reduces glucose levels as the primary target. As previously discussed, cystic fibrosis causes the loss of cells in the pancreas by replacing healthy tissue with fibrosis and amyloid [97]. 

Secondary to this step, the first phase of insulin secretion diminishes, causing hyperglycemia [98]. It is thought that reduced exocytosis affects the production of enough insulin to cover the necessities or lessen the intensity of the insulin peak. It causes postprandial hyperglycemia, leading to diabetes over time. Insulin resistance encountered in type 2 diabetes represents an alternative pathway leading to glucose abnormalities. Hyperglycemia influences the insulin-sensitive channels on cell surfaces, significantly decreasing the abundance of glucose transporters, specifically glucose transporter type 4 [93]. The importance of the transporter is to easily diffuse glucose in muscle and adipose tissue. As an immediate effect, insulin resistance is triggered and accentuated. As a hallmark of cystic fibrosis, glycemic fluctuation is often encountered, accentuated by disease evolution and complication worsening, especially respiratory exacerbations, treatment, hormonal imbalances, and nutritional status. Less muscular and adipose tissues will make stocking glucose impossible [93,99].

### 5.5. Oxidative Stress in CFRD

Cystic fibrosis-associated diabetes in individuals diagnosed with cystic fibrosis manifests as a stand-alone variant. CFRD development is intricately associated with oxidative stress [23]. Maintaining cellular functioning under normal conditions relies on the delicate equilibrium between reactive oxygen species (ROS) and their counteraction by antioxidants [100]. Nevertheless, in the context of cystic fibrosis (CF), there is an atypical excessive generation of reactive oxygen species (ROS), which leads to detrimental effects on the pancreatic β-cells responsible for insulin release [101]. The resulting impaired insulin secretion initiates the onset of glucose intolerance, which is a hallmark of CFRD [4]. 

Further aggravating the condition, the chronic inflammation characteristic of CF can amplify oxidative stress, thereby accelerating the progression of CFRD [10,102]. Identifying and quantifying oxidative stress biomarkers in CFRD patients is crucial for understanding the pathophysiology of the disease and informing potential treatment strategies. Elevated concentrations of malondialdehyde (MDA) and protein carbonyls in the plasma have been recognized as dependable indicators of oxidative stress, indicating the occurrence of lipid and protein oxidation, respectively [47]. Furthermore, the diminished concentrations of reduced glutathione (GSH) observed in individuals with cystic fibrosis-related diabetes (CFRD) highlight the degraded condition of the body’s antioxidant mechanisms [27]. The development of improved detection methods has also facilitated the discovery of novel biomarkers, including F2-isoprostanes and 8-hydroxy-2’-deoxyguanosine (8-OHdG) [102,103]. 

In their study, Ntimbane et al. examined the levels of blood glutathione and 4-hydroxynonenal-protein adducts (HNE-P), urine 1,4-dihydroxy nonane-mercapturic acid conjugate (DHN-MA), and plasma fatty acid (FA) in a cohort of young patients (aged 10–18) following an oral glucose tolerance test (OGTT). They concluded that children with cystic fibrosis displayed higher oxidative stress and poorer glucose metabolism. Nutritional recommendations may help to postpone the onset of CFRD [23]. In a separate investigation carried out by Ntimbane et al., it was determined that the presence of a faulty CFTR protein amplified the compromised antioxidant defense mechanism of β-cells, rendering them more susceptible to oxidative stress occurrences [101].

Hunt et al. conducted a study to assess the systemic redox balance and levels of pro-inflammatory cytokines in patients with cystic fibrosis (CF) both before and after undergoing an oral glucose tolerance test (OGTT). When comparing individuals with prediabetes and cystic fibrosis-related diabetes (CFRD) to those with cystic fibrosis (CF) and standard glucose tolerance (NGT), as well as healthy controls (HC), it was shown that the baseline systemic redox potential of the former group was significantly more oxidized. The glucose administration resulted in a significant and notable increase in systemic oxidation two hours later in all groups with cystic fibrosis, including those with standard glucose tolerance, prediabetes, and cystic fibrosis-related diabetes. The redox imbalance observed at the two-hour mark was consistent across all three groups of individuals with cystic fibrosis and did not show any correlation with the severity of hyperglycemia. In summary, the study conducted by Hunt et al. yielded the finding that a notable association existed between heightened systemic oxidation and diminished insulin secretion [104].

### 5.6. Screening and Diagnosis

From the age of 10, screening is recommended for all cystic fibrosis children [89,105]. Furthermore, patients that should be screened for CFRD include individuals with pulmonary exacerbation under glucocorticoid and antibiotic therapy, enteral or parenteral nutrition support, and undergoing transplantation. The screening and diagnostic methods for cystic fibrosis-related diabetes (CFRD) include the oral glucose tolerance test (considered the gold standard), hemoglobin A1c, fasting plasma glucose, random blood glucose testing, and glucose monitoring [89]. Although other indicators such as fructosamine, glycated albumin, and 1,5-anhydroglucitol are utilized to evaluate glycemic control instead of hemoglobin A1c, they are not indicated for the screening and diagnosis of cystic fibrosis-related diabetes (CFRD) [89,106]. However, studies showed that CGM (continuous glucose monitoring) may be more effective [107,108]. In a study conducted by Taylor–Cousar, continuous glucose monitoring (CGM) showed a higher level of impaired glucose metabolism in adult patients diagnosed with cystic fibrosis (CF) compared to the widely accepted gold standard oral glucose tolerance test (OGTT). Additionally, individuals who experienced the onset of cystic fibrosis-related diabetes (CFRD) within a certain period were effectively detected by continuous glucose monitoring (CGM) when their glucose levels surpassed 200 mg/dL on two separate occasions [108].

Boudreau et al. studied the OGGT results of 152 adult patients without known diabetes. They concluded that alterations in insulin sensitivity were associated with variations in glucose tolerance in adult individuals with CF who had considerably decreased insulin secretion [109].

The study conducted by Chan et al. aimed to investigate the correlation between continuous glucose monitoring (CGM) and estimations of β-cell function generated from oral glucose tolerance tests (OGTT). Additionally, the researchers wanted to assess the potential of CGM as a screening tool for prediabetes and cystic fibrosis-related diabetes (CFRD) as defined by OGTT. The researchers’ investigation concluded a negative correlation between increased glycemic fluctuation measured by continuous glucose monitoring (CGM) and β-cell function. Nevertheless, the performance of continuous glucose monitoring (CGM) was inaccurately differentiating between patients diagnosed with oral glucose tolerance test (OGTT)-defined cystic fibrosis-related diabetes (CFRD) and those without CFRD, as well as individuals with prediabetes [110].

### 5.7. CF Exacerbations Treatment and the Effect on Blood Glucose

Steroid use (typically prednisolone or methylprednisolone) is prevalent in CF patients and may cause transitory hyperglycemia or decrease glucose control in those with pre-existing CFRD. National guidelines recommend more frequent CBG monitoring and the use of insulin to treat hyperglycemia [111,112].

## 6. Cystic Fibrosis Modulator Therapy and the Effect It Has on Glucose Metabolism

CFTR modulators are small-molecule medicines that bind to the CFTR protein with precise site affinity during or after protein processing. The availability of CFTR protein at the cell surface may eventually be increased by several therapeutic approaches. Potentiators, correctors, read-through agents, amplifiers, and stabilizers represent several options. Potentiators and correctors are clinically available, while work is currently being carried out on developing other categories of molecules [113].

**Figure 2:**
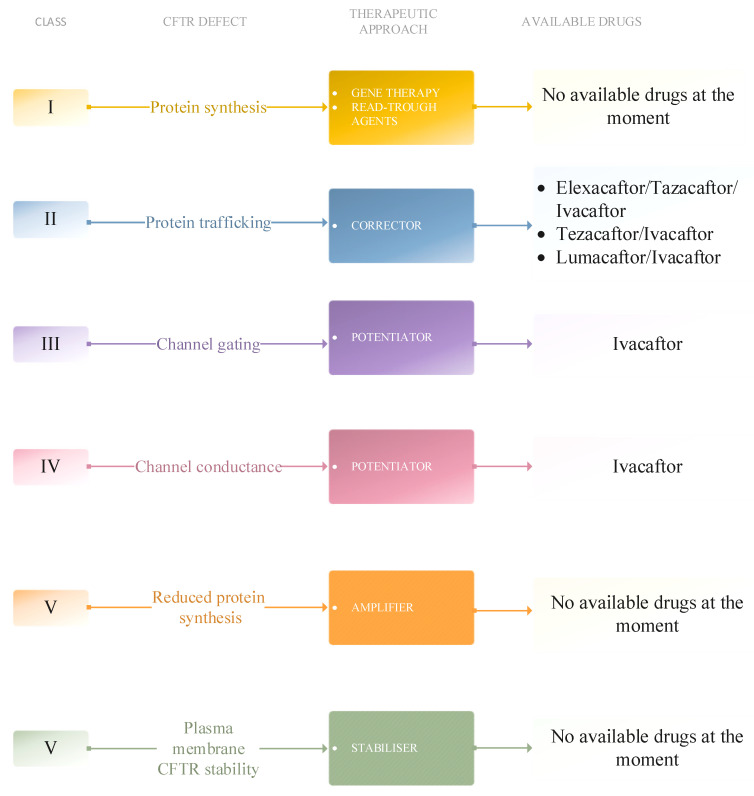
Available therapeutic approaches for each CFTR mutation class at the moment [113,114,115].

The licensing of certain CFTR gene variations for new CFTR modulator therapy in individuals with cystic fibrosis has been supported by clinical research and in vitro test findings [114]. Modulators are designed to target the fundamental anomaly and amplify the functionality of the cystic fibrosis transmembrane conductance regulator (CFTR). The utilization of either monotherapy or a combination of modulators is determined by the specific genotype and type of CFTR disease-causing mutations found in an individual [115] (Figure 2). 

CFTR mutations present six different classes, each based on the cause of dysfunction. Furthermore, patients with CFTR mutations belonging to the first, second, and third classes tend to develop more severe symptoms due to the complexity of protein defects [29].

Ivacaftor, the initial modulator licensed for clinical use in 2012, works as a potentiator by binding to the CFTR protein. 

This interaction leads to an extension in the duration that the mature protein channel remains open, hence augmenting ion transport efficiency across the epithelial cell membrane [113,116,117]. Ivacaftor can be used as a single-agent CFTR modulator, but only a small number of patients are eligible for this therapy [113].

There is evidence that CFTR modulator therapy impacts glucose metabolism [114]. Christian et al. studied the case of a patient diagnosed with CFRD at age 20 who showed improved glycemic control after initiating Ivacaftor [118]. Furthermore, in a study by Gaines et al., 4 out of 14 CFRD patients who were taking Ivacaftor alone or in combination therapy stopped using insulin, and one of the individuals drastically reduced the pre-prandial insulin doses [119]. 

Improved nutritional status, better lung function, better CFRD status, and lower *Pseudomonas aeruginosa* prevalence were observed by Volkova et al., who analyzed data from national UK and US registries [120]. Ivacaftor treatment also improved insulin secretion in a study where Tsabari et al. investigated two cystic fibrosis siblings with impaired insulin secretion. After 16 weeks of treatment, the subjects had improved insulin secretion [121]. Dagan et al. observed eight cystic fibrosis patients who underwent Ivacaftor therapy for one year and showed an improvement in pulmonary function, nutritional status, and glucose tolerance [122]. A notable prevalence of abnormal glucose tolerance exists among young children under five years old diagnosed with cystic fibrosis. They exhibit a deficiency in the typical augmentation of insulin secretion observed during early childhood despite experiencing elevated glucose levels. The results of a study indicate that glycemic irregularities manifest at an early stage in individuals with cystic fibrosis, potentially due to inadequate insulin production [123].

The study conducted by Misgault et al. showed that the administration of Lumacaftor/Ivacaftor therapy significantly improved abnormal glucose tolerance during a one-year treatment period [124]. However, glucose tolerance was shown not to be consistently improved by Lumacaftor/Ivacaftor therapy in several other studies [125,126,127,128].

The efficacy of “Triple Therapy” was studied by Lopez et al. in a series of phase 3 clinical trials. It was demonstrated that Elexacaftor/Tezacaftor/Ivacaftor (ETI) therapy could substantially increase survival in individuals with CF [129]. Furthermore, several authors studied the impacts of that Elexacaftor/Tezacaftor/Ivacaftor therapy on glucose metabolism [130].

Scully and colleagues demonstrated that the commencement of ETI in adult individuals resulted in reduced average glucose levels, standard deviation, percentage of time exceeding 200 mg/dL, and peak sensor glucose. Furthermore, there was an increase in the time elapsed in the target range of 70–180 mg/dL [131]. Korten et al. conducted an observational study to examine the immediate impact of exercise training intervention (ETI) on glucose tolerance in a cohort of young individuals diagnosed with cystic fibrosis (CF). ETI therapy has been determined to benefit endocrine pancreatic function and can potentially prevent or delay the onset of cystic fibrosis-related diabetes (CFRD) [132]. The study conducted by Steinack et al. found evidence suggesting that the administration of the triple CFTR modulator may be associated with a potential enhancement in glucose tolerance among adult persons diagnosed with CF and possessing at least a single variant of the F508de mutation. Notably, this improvement in glucose tolerance was observed without any concurrent elevation in insulin production [133].

In their study, Chan et al. compared the outcomes in 20 individuals, including kids and adults. These individuals underwent a frequently sampled oral glucose tolerance test (OGTT) both before and after ETI medication administration, which showed an increase in BMI z-scores and measures of insulin resistance and secretion during the initial year after ETI commencement. However, no modification was observed in the adjusted function of β-cell for insulin sensitivity [134]. Table 2 contains a summary of the abovementioned investigations.

## 7. Conclusions

The evolving understanding of cystic fibrosis-related diabetes (CFRD) underscores the importance of unraveling the intricate interplay between oxidative stress, inflammation, and insulin secretion in its pathogenesis. The research emphasizes that managing CFRD extends beyond merely controlling blood sugar levels and involves addressing the underlying oxidative stress and inflammation. The promising role of lifestyle modifications such as regular exercise and a balanced diet, in conjunction with optimal insulin dosing, shows that a holistic approach can significantly reduce the impacts of this condition.

The lack of a relationship between initial insulin doses and oxidative stress highlights the complexity of CFRD and the need for multi-dimensional strategies in its management. Further research is required to design interventions that comprehensively address these elements. This presents an opportunity for a paradigm shift in CFRD management-transitioning from a primarily glucose-centric perspective to an approach that emphasizes oxidative stress modulation, inflammation control, and improved insulin sensitivity. 

This innovative direction promises a more personalized and effective treatment regimen, offering a brighter future for individuals living with CFRD.

## Figures and Tables

**Table 1:** Oxidative stress biomarkers analyzed in different types of diabetes [21,23,24,27].

Reference	Point of Interest	Subjects/Methods	Parameter of Interest	Findings
Colomo et al. [21]	T1DM	54 T1DM patients (7–16 years old)	Measurement of 8-iso-prostaglandin F2 alpha in 24-h urine	High urinary excretion of 8-iso-prostaglandin F2 alpha in T1D individuals
Ntimbane et al. [23]	Cystic fibrosis-related diabetes	31 CF patients aged 10–18 years that underwent OGTT	Glutathione 4-hydroxynonenal-protein adducts Urine 1,4-dihydroxynonane-mercapturic acid Conjugated plasma fatty acid	6% of patients had CFRD -HNE-P Important values obtained in patients with diabetes: 42% had IGT 52% had NGT 2-h blood glucose correlated positively with HNE-P and negatively with DHN-MA Glutathione levels were not significantly different between groups
Morandi et al. [24]	T1DM	412 pediatric and young adults with T1DM (3.6–23.5 years old) compared to 138 healthy children/adolescents (1.2–19.2 years old)	Derivatives reactive oxygen metabolites (D-ROMS)	Higher levels of D-ROMs in children/adolescents with T1DM vs. healthy children
Grabia et al. [27]	T1DM	168 patients with T1DM and healthy individuals aged 10–17 years; 103 children and adolescents were diagnosed with T1DM	Arsenic Glutathione peroxidase Malondialdehyde Superoxide dismutase Total antioxidant status Oxidative stress index Catalase Mercury Cadmium Copper/zinc ratio Chromium Lead Selenium Copper Zinc	T1DM children had higher values Cu/Zn ratio, malondialdehyde, total antixoidant status, and oxidative stress index Lower values were found for copper, zinc, superoxide dismutase, and catalase

Abbreviations: T1DM type 1 diabetes, D-ROMS Derivatives reactive oxygen metabolites, IGT: impaired glucose tolerance, NGT: normal glucose tolerance, OGTT oral glucose tolerance test, HNE4 hydroxynonenal, DHNGra dihydroxynonane-mercapturic acid.

**Table 2:** Therapeutic approaches for CFTR.

Reference	Subjects	Studied Modulators	Conclusions
Christian et al., 2019 [118]	A male individual, aged 34, who has been clinically diagnosed with cystic fibrosis-related diabetes (CFRD)	Ivacaftor	Enhanced glycemic control subsequent to the commencement of Ivacaftor administration
Gaines et al., 2021 [119]	Out of a total of 69 adult patients diagnosed with cystic fibrosis (CF), it was found that 31 individuals developed cystic fibrosis-related diabetes (CFRD), with 14 of them receiving CTFR modulator therapy	2 Ivacaftor alone 8 Ivacaftor and lumacaftor 4 Ivacaftor and tezacaftor	4 patients discontinued the use of insulin 1 patient transitioned from administering pre-prandial insulin 3 times daily to use insulin once weekly
Tsabari et al., 2016 [121]	2 sibling patients diagnosed with cystic fibrosis (CF) who possess the S549R gating mutation	Ivacaftor	The insulin secretion pattern was enhanced primarily by augmentation of early insulin secretion in the first phase
Dagan et al., 2017 [122]	8 individuals with cystic fibrosis in Israel who were found to possess the p.Ser549Arg (S549R) mutation	Ivacaftor	The augmentation of CFTR functionality The enhancement of all parameters pertaining to pulmonary function testing Enhanced nutritional condition Enhanced glucose tolerance
Misgault et al., 2020 [124]	40 patientsA total of 78% of the patients exhibited glucose intolerance, whereas 22% of the patients had diabetes at the beginning of the study	Lumacaftor/Ivacaftor	Improved abnormal glucose tolerance after 1 year of treatment
Thomassen et al., 2018 [125]	5 Phe508del-homozygous CF patients	Lumacaftor/Ivacaftor	An improvement of 2-h glucose levels in 3 patients Worsening of 2-h glucose in 2 patients
Moheet et al., 2021 [127]	A total of 39 individuals exhibiting homozygosity for the F508del mutation were included in the study	Lumacaftor/Ivacaftor	There was no observed enhancement in the production of insulin or improvement in glucose tolerance
Colombo et al., 2021 [128]	13 patients homozygous for the F508del mutation and 13 controls A total of 26 individuals were included in the study, consisting of 13 patients who were	Lumacaftor or Ivacaftor	There is a lack of empirical support indicating any enhancement in glucose tolerance homozygous for the F508del mutation and 13 control subjects
Scully et al., 2022 [131]	34 adults with CF and at least 1 F508del CFTR mutation	Elexacaftor/Tezacaftor/Ivacaftor	23 individuals completed the study The research team at ETI conducted an analysis and saw a reduction in the mean glucose levels, standard deviation, percentage of time spent above the threshold of 200 mg/dL, and the highest recorded sensor glucose readings The ETI study observed an increase in the percentage of time spent within the target range of 70–180 mg/dL
Korten et al., 2022 [132]	16 adolescents	Elexacaftor/Tezacaftor/Ivacaftor	OGTT was improved Glucose levels improved at 60, 90, 120 min Fasting glucose and CGM measures were not modified
Steinack et al., 2023 [133]	The study included a total of 33 individuals, with an average age of 27.8 ± 6.3 years; among these patients, 73% were male and 64% were homozygous for the F508del mutation	Elexacaftor/Tezacaftor/Ivacaftor	A total of 16 individuals, accounting for 48.5% of the sample, exhibited an improvement in their glucose tolerance category 13 (39.4%) remained unchanged 4 presented a clinical decline The glycemia levels for the 60, 90, and 120-min oral glucose tolerance test (OGTT) were seen to be dramatically reduced HbA1c levels significantly improved
Chan et al., 2022 [134]	20 youth and adults	Elexacaftor/Tezacaftor/Ivacaftor	The BMI z-score showed a rise from 0.3 (−0.3, 0.8) to 0.8 (0.4, 1.5) during the course of the visits There were no statistically significant differences observed in glucose tolerance, glucose area under the curve, or frequently collected oral glucose tolerance test (OGTT) glucose values The median C-peptide index exhibited a significant rise, rising from 5.7 (4.1, 8.3) to 8.8 (5.5, 10.8). HOMA2 IR increased C-peptide oral disposition index did not change HbA1c decreased from 5.5% (5.5, 5.8) to 5.4% (5.2, 5.6) CGM variables did not change

Abbreviations: Cystic fibrosis-related diabetes (CFRD), Cystic fibrosis (CF), Exercise training intervention (ETI), oral glucose tolerance test (OGTT), glycosylated hemoglobin (HbA1c), Body Mass Index (BMI), Model assessment of insulin Resistance (HOMA), Continuous glycemic monitoring (CGM).
